# Logistic modeling to predict the minimum inhibitory concentration (MIC) of olive leaf extract (OLE) against *Listeria monocytogenes*

**DOI:** 10.1371/journal.pone.0263359

**Published:** 2022-01-28

**Authors:** Renjie Du, Yuejun Qu, Min Zhao, Yanhong Liu, Phoebe X. Qi, Xingbin Sun

**Affiliations:** 1 College of Life Science, Northeast Forestry University, Harbin, Heilongjiang Province, People’s Republic of China; 2 Mudanjiang Branch of Heilongjiang Academy of Forestry Science, Mudanjiang, Heilongjiang Province, People’s Republic of China; 3 Molecular Characterization of Foodborne Pathogens Research Unit, Eastern Regional Research Center, Agricultural Research Service, United States Department of Agriculture, Wyndmoor, Pennsylvania, United States of America; 4 Dairy and Functional Foods Research Unit, Eastern Regional Research Center, Agricultural Research Service, United States Department of Agriculture, Wyndmoor, Pennsylvania, United States of America; Spanish Council for Scientific Research (CSIC), SPAIN

## Abstract

Olive leaf extract (OLE) has been increasingly recognized as a natural and effective antimicrobial against a host of foodborne pathogens. This study attempts to predict the minimum inhibitory concentration (MIC) of OLE against *Listeria monocytogenes* F2365 by utilizing the asymptotic deceleration point (PDA) in a logistic model (LM), namely MIC-PDA. The experimental data obtained from the inhibitory rate (IR) versus OLE concentration against *L*. *monocytogenes* were sufficiently fitted (*R*^2^ = 0.88957). Five significant critical points were derived by taking the multi-order derivatives of the LM function: the inflection point (PI), the maximum acceleration point (PAM), the maximum deceleration point (PDM), the absolute acceleration point (PAA), and the asymptotic deceleration point (PDA). The PDA ([OLE] = 37.055 mg/mL) was employed to approximate the MIC-PDA. This MIC value was decreased by over 42% compared to the experimental MIC of 64.0 mg/mL, obtained using the conventional 2-fold dilution method (*i*.*e*., MIC-2fold). The accuracy of MIC-PDA was evaluated by an *in vitro L*. *monocytogenes* growth inhibition assay. Finally, the logistic modeling method was independently validated using our previously published inhibition data of OLE against the growths of *Escherichia coli* O157:H7 and *Salmonella enteritidis*. The MIC-PDA (for [OLE]) values were estimated to be 41.083 and 35.313 mg/mL, respectively, compared to the experimental value of 62.5 mg/mL. Taken together, MIC-PDA, as estimated from the logistic modeling, holds the potential to shorten the time and reduce cost when OLE is used as an antimicrobial in the food industry.

## Introduction

Bacterial minimum inhibitory concentration (MIC) is defined as the lowest concentration of the antimicrobial agent assayed to prevent the visible growth of a bacterium after overnight incubation [1]. MIC values are used to assess the susceptibilities of bacteria to a particular drug or an antimicrobial compound [2]. They are commonly obtained by 2-fold serial dilutions of the antimicrobial agent in broth and assayed using 96-well microtiter plates, *i*.*e*., the MIC-2fold, for example, in the case of olive leaf extract (OLE) against *Listeria monocytogenes* [3], a major foodborne pathogen. However, the MIC-2fold method is usually time-consuming and costly. It tends to overestimate the MIC values [4,5], caused by the large value-span between the last two concentration points (at the higher end of the data range).

OLE is derived from the leaves of olive trees. It has been used in traditional medicine for its various health benefits due to its high content of phenolic compounds [6]. The potential application of OLE has been recently recognized as a natural antimicrobial agent [3,7,8], either used as an additive in foods [9] or more commonly incorporated into food packaging, such as films [10–13] to enhance food safety. The parameters that control the qualities of these antimicrobial films, such as thickness, density, *etc*., are closely correlated with the MIC value in the food packaging industry [14], implying a higher MIC value would lead to a higher cost of the product. Most importantly, high OLE levels (> 20 mg/100 g) could cause strong bitterness and pungent perception in foods [15]. Masking agents, such as sugar or salt, are often added to mask these undesirable flavors, introducing unnecessarily elevated amounts of sugar or sodium. Extensive research has established a probable link between over-consumption of sugar and sodium and increased risk in cardiometabolic disease, hypertension, insulin resistance, fatty liver, obesity, and dental caries [16–18]. Therefore, a higher OLE level resulted from a greater MIC (than necessary) would cause adverse nutritional and health effects. Consequently, finding a practical and reasonable MIC value is critical for the viable and broad applications of OLE to be realized and expanded in the food industry.

According to Liu *et al*. [3,8], the inhibitory rate (IR) for OLE against *L*. *monocytogenes* resembled a sigmoidal growth trend as the OLE concentration increased, closely following a logistic model (LM) [19] rather than an exponential correlation. Although the logistic function and its variants have found uses to address microbial growth dynamics in a habitat of finite resources in food [20,21], referred to as the Verhulst model, the significance of its derivatives remains to be examined and exploited by the microbiology community. They (the logistic function and its derivatives) have been utilized in other research fields, including the mathematical characterization and understanding of the recent COVID-19 outbreak in Italy [22]. They have also been applied to evaluate the callus growth kinetics and the accumulation of secondary metabolites of *Bletilla striata*, a traditional Chinese medicinal plant [23], and simulate the growth of *Centrolobium tomentosum* fruits in Brazil for economic reasons [24]. Five meaningful critical points associated with growth kinetics have been reported for LM function: the absolute acceleration point (PAA), the maximum acceleration point (PAM), the inflection point (PI), the maximum deceleration point (PDM), and the asymptotic deceleration point (PDA) [25,26]. These mathematically derived critical points separate the LM (0 ≤ *x* < ∞) into several phases and can be applied to describe the stages of varying biological significance (differential growth rate, acceleration, and deceleration) [26]. Among these points, the PDA can be of particular relevance and holds the potential to be used to evaluate the level to which the bacterial growth reaches sufficiently close to the asymptote (LM generally has an upper horizontal asymptote), so that the following experimental data points can be ignored [24,25].

Thus far, the applications of LM have been primarily limited to investigating the behavior of the growth function (population, fruit growth, callus, or virus proliferation) as a function of time [22–25]. To our best knowledge, correlating these critical points with a broad range of biological observations, including MIC determination, remains primarily unreported in the literature. In this work, we explore the feasibility of investigating the inhibitory behavior of olive leaf extract (OLE) exerted against *L*. *monocytogenes* F2365 using a logistic function and the calculated critical points.

The objectives of this work were to: (1) predict a MIC value for OLE against *L*. *monocytogenes* F2365 using the asymptotic deceleration point (PDA) derived from the LM, *i*.*e*., MIC-PDA; (2) delineate the underlying mathematical significance for the inhibitory process; and (3) evaluate the accuracy of MIC-PDA by conducting conventional inhibitory assays at similar OLE concentration.

## Materials and methods

### Materials

*L*. *monocytogenes* strain F2365 was from the Eastern Regional Research Center (ERRC) collections. Brain Heart Infusion (BHI) broth was purchased from Sigma-Aldrich Inc. (St. Louis, MO). Olive leaf extract (OLE) was a gift from EuroMed, Inc. (Barcelona, Spain).

### Inhibitory assays of OLE against *L*. *monocytogenes*

A single colony of *L*. *monocytogenes* F2365 was inoculated in 5 ml Brain Heart Infusion (BHI) broth and incubated overnight at 37°C with controlled agitation at 200 rpm. A 1:1000 dilution of the overnight culture with BHI broth was used for the inhibition study. The inhibitory activities of OLE against *L*. *monocytogenes* F2365 were assessed by a two-fold serial broth dilution method using 96-well microtiter plates, as described previously [3]. Briefly, eight OLE concentrations (128.0, 64.0, 32.0, 16.0, 8.0, 4.0, 2.0, and 1.0 mg/ml) were generated by two-fold serial dilutions using BHI broth. Fifty microliters (50 μL) of OLE at each concentration and 50 μL of diluted *L*. *monocytogenes* F2365 overnight culture, total 100 μL, were mixed into each well of the plate. After 24 h incubation, the OD_600_ was measured using a spectrophotometric microplate reader (BioTek Instruments, Inc., Winooski, VT) at 37°C with 0.05 linear agitation, as programed by the Gen 5 software (v. 3.00.19). Eight replicates (on the plate) were set up for each OLE concentration condition.

### Inhibitory rate (IR) of OLE against *L*. *monocytogenes*

The inhibitory rate (IR) of OLE against *L*. *monocytogenes* was calculated using the OD_600_ data at varying OLE concentrations from our previous work [27]. Some modifications were made base on the published method [28], as indicated below in Eq 1:

$$\text{IR}=\left(1-\frac{{\text{OD}}_{600}\left(\text{EG}\right)-{\text{OD}}_{600}\left(\text{OLE}\right)}{{\text{OD}}_{600}\left(\text{PC}\right)-{\text{OD}}_{600}\left(\text{NC}\right)}\right)\times 100\text{\%}$$ (1)

Where PC, NC, and OLE stand for the positive control (*L*. *monocytogenes* overnight culture), the negative control (BHI broth only), and OLE only (at each corresponding OLE concentration, specified in Section “Inhibitory assays of OLE against *L*. *monocytogenes*”) absorbance value, respectively. EG denotes the experimental group (at each OLE concentration).

### Logistic regression modeling and calculations of critical points

A scattered plot of the IR values (from Eq 1) versus OLE concentration was generated and then fitted with a logistic function, Eq 2 [25] with slight modifications using Origin (v. 2019b, OriginLab Inc.) software.


$$y=\frac{\alpha }{1+exp\left(-\beta -\gamma x\right)}$$ (2)

Where *x* stands for OLE concentration ([OLE]), *y*, the corresponding IR value, and α, the asymptotic value (100.00% in this case). β and γ are the parameters fitted for IR in a logistic relationship with OLE concentration. The correlation of determination, *R*^2^, was calculated during the regression fitting by Origin software.

Taking first-, second-, third-, and fourth-order derivatives of Eq 2 yielded critical points and their values for the IR function versus changing OLE concentration. These critical points were termed as inflection point (PI), maximum acceleration point (PAM), maximum deceleration point (PDM), absolute acceleration point (PAA), and asymptotic deceleration point (PDA), similar to those of the *Eucalyptus camaldulensis* Dehnh. tree growth model [25]. At these points, the logistic function (IR) gradient (derivative) is equal to zero as a function of change in OLE concentration. The first-order derivative function of Eq 2, *y’*, returned no critical point as it is also a logistic function, which never reaches zero (as *x* →∞). The second-order derivative function, *y’’*, produced the inflection point (PI, Eq 3) with the following values:

$$x_{\text{PI}}=-\frac{\beta }{\gamma };\;y_{\text{PI}}=\frac{\alpha }{2}$$ (3)

The third-order derivative, *y‴*, prompted values for PAM and PDM, calculated below (Eqs 4 and 5):

$$x_{\text{PAM}}=-\frac{Ln\left(2+\sqrt{3}\right)+\beta }{\gamma };\;y_{\text{PAM}}=\frac{\alpha }{3+\sqrt{3}}$$ (4)

$$x_{\text{PDM}}=-\frac{Ln\left(2-\sqrt{3}\right)+\beta }{\gamma };\;y_{\text{PDM}}=\frac{\alpha }{3-\sqrt{3}}$$ (5)

The fourth-order derivative, *y‴’*, generated PI, PAA, and PDA values, as detailed in Eqs 3, 6 and 7, respectively.


$$x_{\text{PAA}}=-\frac{Ln\left(5+2\sqrt{6}\right)+\beta }{\gamma };\;y_{\text{PAA}}=\frac{\alpha }{6+2\sqrt{6}}$$ (6)

$$x_{\text{PDA}}=-\frac{Ln\left(5-2\sqrt{6}\right)+\beta }{\gamma };\;y_{\text{PDA}}=\frac{\alpha }{6-2\sqrt{6}}$$ (7)

### Inhibition assay using OLE concentration close to MIC-PDA

The inhibitory activity of OLE against *L*. *monocytogenes* F2365 at close to the MIC-PDA (37.055 mg/mL) was evaluated using the assay as described above, in Sections “Inhibitory assays of OLE against *L*. *monocytogenes*” and “Inhibitory rate (IR) of OLE against *L*. *monocytogenes*”. Briefly, 50 μL of diluted (74.0 mg/mL) from the OLE stock solution (dissolved in BHI at 250 mg/mL) and 50 μL *L*. *monocytogenes* F2365 (diluted 1000X in BHI) overnight culture were mixed into each well of the plate, making the final OLE concentration ~37.0 mg/mL in each well. Total eight wells (at the same OLE concentration) were used. The positive control contained 50 μL of *L*. *monocytogenes* overnight culture and 50 μL BHI broth (instead of OLE). The negative control included 100 μL BHI broth only. The blank was a mixture of equal volume (50 μL) of OLE (at 74.0 mg/mL) and BHI. The OD_600_ was measured after 24 h incubation, as described above, and the IR was calculated using Eq 1.

### Statistical analysis

Statistical analysis was performed using Excel (v. 365) and Origin (v. 2019b, OriginLab Inc.) software package where P < 0.05 was considered significant.

## Results and discussion

### Experimental data of IR versus OLE concentration

**Fig 1:**
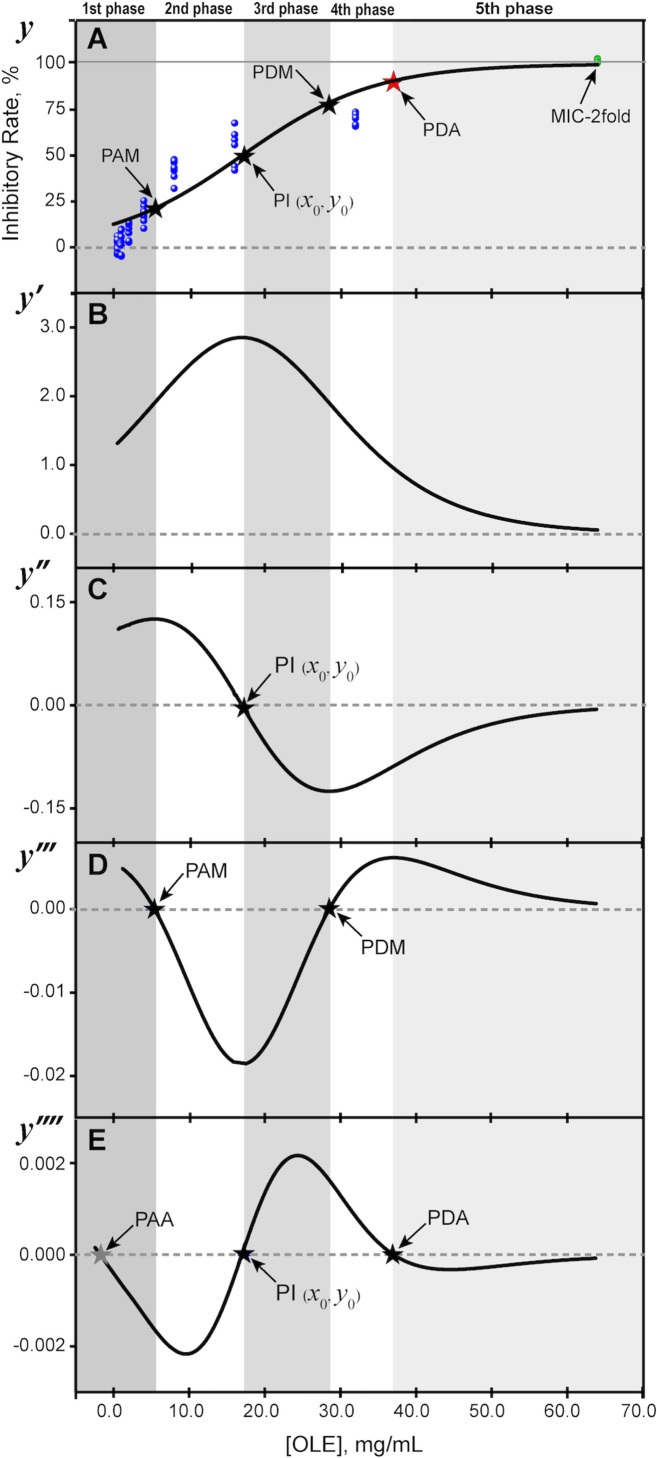
Logistic modeling (LM) of inhibitory rates (IR) versus OLE concentration against *L*. *monocytogenes* F2365. (A) the experimental data (blue balls) and the logistic regression curve (solid black line, β = -1.9366; γ = 0.11413, and *R*^2^ = 0.88957); (B) the first-order derivative (*y’*); (C) the second-order derivative (*y’’*) generated PI, *x*_0_ (= 16.968 mg/mL) and *y*_0_ (= 50.000%); (D) the third-order derivative (*y‴*) produced the maximum acceleration point (PAM (5.4293 mg/mL, 21.132%)) and the maximum deceleration point (PDM (28.507 mg/mL, 78.868%)); (E) the fourth-order derivative (*y‴’*) yielded inflection point (PI (16.968 mg/mL, 50.000%)) and asymptotic deceleration point (PDA (37.055 mg/mL, 90.825%)).

Fig 1A shows the experimental data obtained for IR as a function of OLE concentration using the conventional 2-fold dilution method, agreeing with the result of Liu *et al*. (2017 and 2018). In addition, the data approached a sigmoidal-shaped curve rather than a linear correlation, suggesting that the distribution of IR versus OLE concentration in the inhibitory activities against *L*. *monocytogenes* resembled a logistic function. Subsequently, a logistic regression procedure was employed to fit the experimental data to depict and predict the critical points and phases of the inhibitory process.

The β and γ values in Eq 2 were estimated to be -1.9366 and 0.11413, respectively, by the logistic regression routine in Origin software. The resulting *R*^2^ (correlation of determination) value was 0.88957, indicating a reasonably close fit [29] between the experimental data and the logistic model (LM) used. This established mathematical relationship makes it possible to estimate the OLE concentration reliably against *L*. *monocytogenes* at a desired IR value and vice versa.

### Critical points of the logistic function

Fig 1B–1E show the first-, second-, third-, and fourth-order derivatives of the logistic function (Eq 2). The critical points, designated as PI (*x*_0_, *y*_0_), PAM (*x*_PAM_, *y*_PAM_), PDM (*x*_PDM_, *y*_PDM_), PAA (*x*_PAA_, *y*_PAA_), and PDA (*x*_PDA_, *y*_PDA_), are as indicated. The second-, third-, and fourth-order derivatives yielded critical values of the OLE concentrations (*x*-axis) to be 16.968, 5.4293, 28.507, -3.1178, and 37.055 mg/mL, respectively. The corresponding IR values (*y*-axis) were calculated to be 50.000, 21.132, 78.868, 9.1752, and 90.825%. The PAA point was of little significance for a negative value (-3.1178 mg/mL) was attained in this work.

The remaining four critical points represented five phases of the changes that occurred in IR as affected by increasing OLE concentration. As shown in Fig 1B (*y’*), the first-order derivative produced no critical point. The second-order derivative (*y’’*, Fig 1C) returned the critical point PI (*x*_0_, *y*_0_), *i*.*e*., the inflection point, corresponding to the OLE concentration (16.968 mg/mL) at half (50.000%) of the total IR. PI signifies the most rapid IR increase at this OLE concentration. After the inflection point, the rising rate of IR declines, approaching the limiting value.

PAM (Fig 1D), the maximum acceleration, and PDM, the maximum deceleration at increasing OLE concentration, were obtained by taking the third-order derivative of Eq (2) (*y‴*, Fig 1D). Their determination revealed that the IR rose rapidly before the PAM value (in the 1^st^ phase), increased gradually from PAM to PI (same as (*x*_0_, *y*_0_)) (the 2^nd^ phase), then declined quickly to PDM (the 3^rd^ phase). The fourth-order derivative (*y‴’*, Fig 1E) identified, once again, the inflection point (PI, the 4^th^ phase), the absolute acceleration point (PAA, also fell in the 1st phase), and the asymptotic deceleration point (PDA) formed the 5th phase. The significance of PDA implied that the IR became increasingly insensitive to the growing OLE concentration (*x* →∞) after this point. The OLE concentration was estimated to be 37.055 mg/mL at the asymptotic deceleration point (PDA), and an IR value of 90.825% was obtained (Eq 7). Therefore, it is reasonable to use PDA as the criterion to predict the minimum inhibitory concentration (MIC) of OLE against *L*. *monocytogenes*.

### Evaluation of MIC-PDA by inhibition assay (MIC-2fold)

To evaluate the accuracy of MIC derived from PDA calculations (MIC-PDA), we performed *in vitro L*. *monocytogenes* growth inhibition assays at the OLE concentration close to the MIC-PDA value (~37 mg/mL). Table 1 compares the inhibitory rate (IR, %) obtained by the two methods. At an identical OLE concentration, the experimental IR (101.8 ± 0.7%) was ~10% higher than the value estimated from PDA (90.825%). We attributed this discrepancy to the experimental errors introduced in the dilutions of OLE and propagated in subsequent assays. Additionally, possible variations in the OD_600_ measurements resulted in an over-estimation of IR (> 100%). Contrarily, the asymptotic value was set at 100.00% (Eq 2) for the calculation of MIC-PDA, strictly limiting the IR values to be below 100.0% as [OLE] →∞.

**Table 1:** Comparison of inhibitory rate (%) against *L*. *monocytogenes* F2365 at similar OLE concentration (MIC-PDA).

[OLE], mg/mL	IR, %	Method
37.055	90.825	PDA estimation
37.0	101.8 ± 0.7	Inhibition assay

Incidentally, the LM (Eq 2) yielded an IR value of 12.602% in the absence of OLE ([OLE] = 0.0), *i*.*e*., the equivalent of the positive control (Eq 1) rather than 0.0%, as determined by the inhibition experiments (Sections “Inhibitory assays of OLE against *L*. *monocytogenes*” and “Experimental data of IR versus OLE concentration”). Non-zero *y* (the IR in this case) is an inherent property of the logistic function. As defined by Eq 2, *y* never reaches 0.0 regardless of the x value unless α is also 0.0, meaning no inhibition effect exists. Nevertheless, this work demonstrated that the logistic modeling procedure was particularly applicable for the presence of an antimicrobial agent ([OLE] > 0.0 mg/mL) to exert a measurable inhibition effect (IR > 0.0%).

### Validation of the logistic modeling method in OLE inhibition of *Escherichia coli* O157:H7 and *Salmonella enteritidis*

To further validate the logistic modeling method, as described in this work, we applied the logistic function (Eq 2) to fit our previously published OLE inhibition data against two Gram-negative strains, *Escherichia coli* O157:H7 and *Salmonella enteritidis*. The resulting parameters (Eqs 3–7) are presented in Table 2, compared to the MIC data obtained by 2-fold dilution inhibition assays [3,8]. Similar to the case of OLE inhibition against *L*. *monocytogenes*, as studied in this work, the PDA-derived MIC values, 41.083 and 35.313 mg/mL, respectively, were significantly lower than the experimentally measured MIC at 62.5 mg/mL. The IR level for these bacterial strains was calculated to be 90.825% when the OLE concentration reached MIC-PDA. The two-fold dilution inhibition assay method appeared to overestimate the MIC in all three bacterial growth scenarios. The logistic modeling reasonably estimated the MIC values for all bacterial strains whose inhibition behavior by OLE followed a logistic function.

**Table 2:** Results from the logistic modeling of OLE inhibition against *Escherichia coli* O157:H7 and *Salmonella enteritidis* using previously published growth inhibition data and comparing with the experimental MIC values* (Liu et al., 2017).

Derived parameters	*E*. *coli* O157:H7	*S*. *enteritidis*
Β	-2.0106	-1.7686
γ	0.10474	0.11500
*R* ^2^	0.98090	0.96621
MIC-PDA (mg/mL, *x*_PDA_)	41.083	35.313
IR (%, *y*_PDA_)	90.825	90.825
MIC* (mg/mL, Liu et al., 2017)	62.5	62.5
IR* (%, Liu et al., 2017)	95	100
IR (%, positive control, when [OLE] = 0.0)	11.809	14.572
PI ((*x*_0_, *y*_0_), (mg/mL, %))	19.196, 50.000	15.379, 50.000
PAM ((*x*_PAM_, *y*_PAM_), (mg/mL, %))	6.6228, 21.132	3.9269, 21.132
PDM ((*x*_PDM_, *y*_PDM_), (mg/mL, %))	31.770, 78.868	26.830, 78.868
PAA ((*x*_PAA_, *y*_PAA_), (mg/mL, %))	-2.6905, 9.1752	-4.5555, 9.1752

The logistic modeling (Eq 2) of the data for *E*. *coli* O157:H7 and *S*. *Enteritidis* was performed by assuming a complete inhibition (α = 100.00%), contrasting our previously experimentally determined IR of 95% for *E*. *coli* O157:H7 [3] compared to 100% for *S*. *Enteritidis* and *L*. *monocytogenes*. Indeed, all derived MIC-PDA, PI (*x*_0_), PAM (*x*), and PAA (*x*) values (Table 2) were higher for *E*. *coli* O157:H7 than those for *S*. *Enteritidis* and *L*. *monocytogenes*. These estimated parameters supported the observations that OLE was less effective in inhibiting *E*. *coli* O157:H7 than against *S*. *Enteritidis* and *L*. *monocytogenes*.

Consistent with the LM results of *L*. *monocytogenes* in Section “Evaluation of MIC-PDA by inhibition assay (MIC-2fold)”, Table 2 showed the IR of 11.809 and 14.572% for *E*. *coli* O157:H7 and *S*. *Enteritidis*, respectively, in the absence of OLE ([OLE] = 0.0 mg/mL), *i*.*e*., the positive control. Once again, Eq 2 and subsequent calculations were more suitable for studying the effective action of an antimicrobial (*e*.*g*., [OLE] > 0.0 mg/mL) against a bacterial strain (when IR > 0.0%).

## Conclusions

A logistic relationship was established (*R*^2^ = 0.88957) for the experimental data of *L*. *monocytogenes* F2364 inhibitory rate (%) as a function of OLE concentration. Taking multi-order derivatives of the logistic function (accomplished by the commercial software package, Origin) yielded four meaningful critical points, *i*.*e*., PI, PAM, PDM, and PAD, and five phases. The asymptotic deceleration point (PAD) proved to be of particular significance as it (MIC-PDA) approximated the minimum inhibitory concentration (MIC) of OLE and agreed reasonably well with the MIC value obtained from the experimental inhibition assays. This work demonstrated combined approaches to yield MIC, *i*.*e*., estimation by taking the derivatives of a logistic function fitted with the inhibition data and direct determination by inhibition essays. We further independently validated the modeling method by applying the logistic function (Eq 2) to previously published data of OLE against two Gram-negative bacterial strains, *Escherichia coli* O157:H7 and *Salmonella enteritidis*. The resulting PDA-derived MIC values (MIC-PDA), 41.083 and 35.313, respectively, were significantly lower than those experimental MIC values, 62.5 mg/mL, similar to the case of OLE inhibition of *L*. *monocytogenes* studied in this work. Moreover, the LM results supported the observation that OLE was less effective against *E*. *coli* O157:H7 than *S*. *enteritidis* and *L*. *monocytogenes*. This logistic modeling procedure may be of particular interest in those cases when the observed variables follow a logistic relationship and help understand the biological events involved.

## Supporting information

S1 Graphical abstract (TIF)
