# Supplementary figures and images for: Logistic modeling to predict the minimum inhibitory concentration (MIC) of olive leaf extract (OLE) against Listeria monocytogenes
Source: PLoS One. 2022 Jan 28;17(1):e0263359. doi: 10.1371/journal.pone.0263359 (PMC8797264; doi:10.1371/journal.pone.0263359)

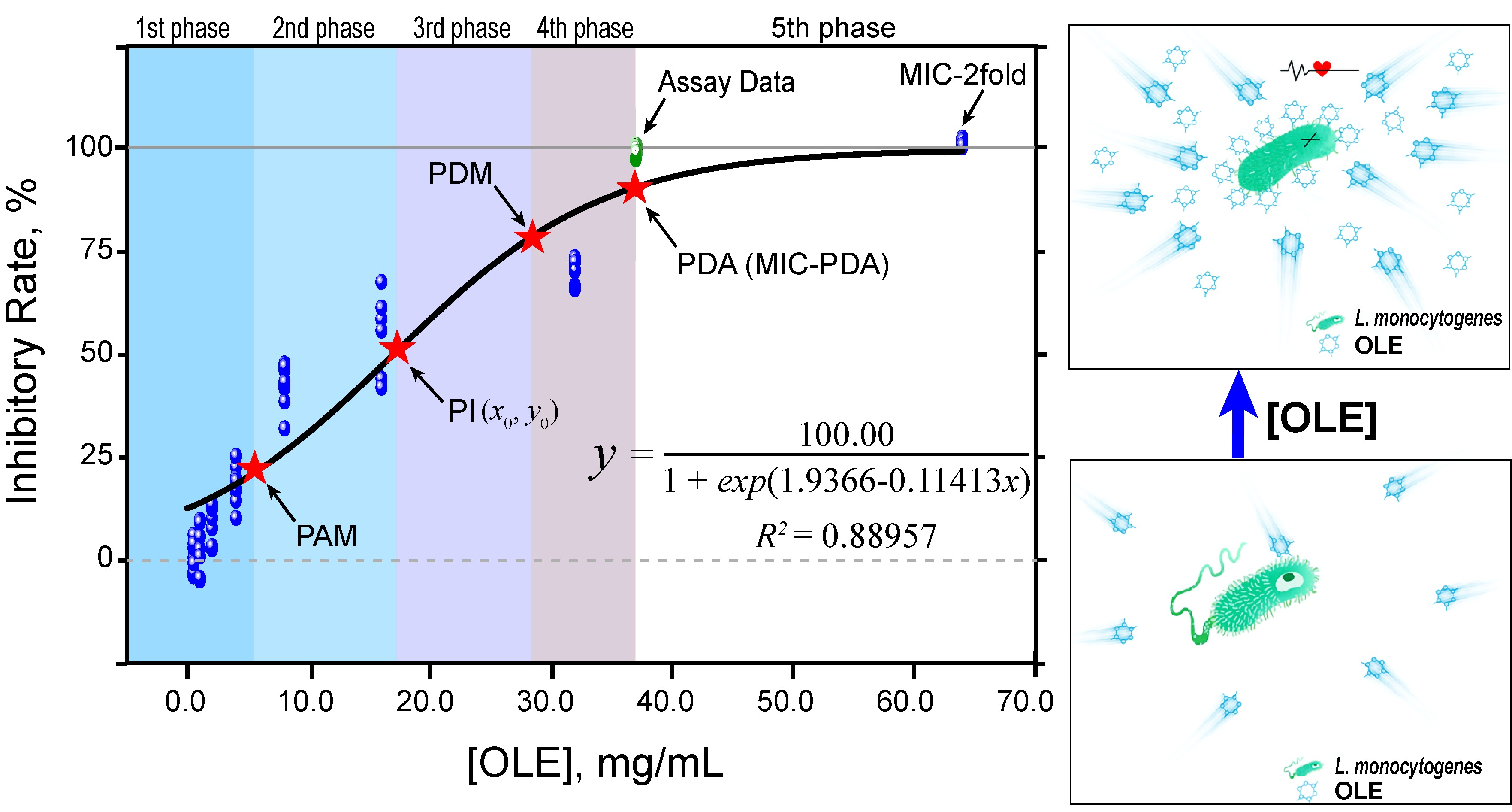

Supplement: S1 Graphical abstract — (TIF) [file pone.0263359.s001.tif]
